# Interpreting the Findings From the Recent PCSK9 Monoclonal Antibody Cardiovascular Outcomes Trials

**DOI:** 10.3389/fcvm.2019.00014

**Published:** 2019-03-06

**Authors:** Nathan D. Wong, Michael D. Shapiro

**Affiliations:** ^1^Heart Disease Prevention Program, Division of Cardiology, University of California, Irvine, Irvine, CA, United States; ^2^Knight Cardiovascular Institute, Oregon Health Sciences University, Portland, OR, United States

**Keywords:** proprotein convertase sibtilisin / kexin type 9 (PCSK9), dyslipidemia, low density lipoprotein-cholesterol (LDL-C), cardiovascular disease, prevention, clinical trials

## Abstract

The recent development of monoclonal antibodies targeted to proprotein convertase subtilisin/kexin type 9 (PCSK9), e.g., PCSK9 inhibitors has revolutionized the landscape of lipid management. Many clinical trials assessing this class have demonstrated remarkable and consistent reductions in low-density lipoprotein-cholesterol. Moreover, the GLAGOV trial demonstrated the efficacy of evolocumab, when added to statin therapy, in reducing the progression of atherosclerosis measured by serial intravascular ultrasound, with the first suggestion of continued benefit down to LDL-C levels of 0.5 mmol/L (20 mg/dL). This trial was followed by the FOURIER Cardiovascular Outcomes trial in more than 27,000 patients with stable atherosclerotic cardiovascular disease (ASCVD) where evolocumab reduced the primary endpoint of atherosclerotic events by 15%, without significant safety differences between treatment groups. Furthermore, subgroup analyses suggested greater benefits seen in those with longer exposure to evolocumab recent acute coronary syndrome, multiple myocardial infarctions, multivessel coronary artery disease, peripheral arterial disease, as well as the subgroup who achieved very low low-density lipoprotein-cholesterol levels of below 0.3 mmol/L (10 mg/dL). Moreover, the EBBINGHAUS substudy demonstrated no differences in objectively measured cognitive function between treatment groups. The SPIRE 2 trial evaluating bococizumab in high-risk patients with baseline LDL-C ≥2.6 mmol/L (100 mg/dL) demonstrated significant atherosclerotic risk reduction, but the trial and further development of the drug was prematurely discontinued due to substantial attenuation of the LDL-C effect over time due to the development of neutralizing antibodies. Finally, the ODYSSEY Cardiovascular Outcomes trial testing alirocumab in subjects with recent (<1 year) acute coronary syndrome demonstrated a 15% relative risk reduction in the primary composite outcome, as well as a significant reduction in total mortality. Greater benefits were noted in those whose LDL-C at baseline was 2.6 mmol/L (100 mg/dL) or greater. These trials collectively demonstrate the added efficacy of PCSK9 inhibitors over moderate and high-intensity statin therapy for unprecedented low-density lipoprotein-cholesterol reduction and incremental ASCVD risk reduction.

Over the past three decades, multiple primary and secondary prevention clinical trials testing the statin drugs have demonstrated the value of low-density lipoprotein (LDL)-cholesterol (LDL-C) lowering in reducing atherosclerotic cardiovascular disease (ASCVD) risk by ~30–40% [and ~20% per mmol/L (~38.7 mg/dL) reduction]. However, this observation implies that the majority of patients, despite effective statin therapy, remain at risk for events. The ASCVD risk that remains despite optimal medical therapy has been termed “residual risk” and has served as the motivation for the development of additional therapies (1). The drivers of residual risk are likely multifactorial as the statin trials largely focused on a single intervention, often comparing a higher to a lower intensity statin, or in the case of older trials, statin compared to placebo. These statin mega-trials did not address other risk factors such as hypertension, diabetes, or cigarette smoking. The IMPROVE-IT trial tested the combination of simvastatin and ezetimibe compared to simvastatin alone. The combination therapy group achieved lower LDL-C levels (median time-weighted LDL-C of 1.4 mmol/L (54 mg/dL) vs. 1.8 mmol/L (70 mg/dL) in the simvastatin monotherapy group) and a modest yet statistically significant reduction in risk beyond the statin monotherapy arm (2). The question remained, however, whether further reduction of ASCVD risk could be achieved from even greater LDL-C lowering to levels not previously observed in major clinical trials.

The availability of two proprotein convertase subtilisin-like kexin type 9 (PCSK9) monoclonal antibody therapies, alirocumab and evolocumab to treat dyslipidemia in persons with ASCVD or familial hypercholesterolemia requiring additional LDL-C reduction despite optimization of lifestyle changes and maximally tolerated statin therapy, has revolutionized the landscape for treatment of dyslipidemia (3). Moreover, the ensuing pivotal cardiovascular outcomes trials of these products over the past 2 years have provided a means to lower residual ASCVD risk and has confirmed the notion that the lowest LDL-C in high-risk patients is best. This review will discuss the findings and implications of these and other recent outcomes trials of PCSK9 monoclonal antibody therapy to better understand lessons learned from these trials and patient subgroups who may benefit most from these agents (Table 1).

**Table 1:** Summary of PCSK9 cardiovascular outcome studies.

**Name of trial**	**Citation**	**Study population**	Intervention^*^	Control^*^	**Follow-up time**	**Primary endpoint**	**Secondary endpoint(s)/subgroup analyses**
GLAGOV	(4)	ASCVD	Evolocumab 420 mg	Placebo	18 months	0.95% decrease vs. 0.05% increase in plaque atheroma volume	Total atheroma volume (−3.6 mm^3^ vs. −0.8 mm^3^; p = 0.04)
FOURIER	(5–13)	ASCVD	Evolocumab 140 or 420 mg	Placebo	2.2 year	15% reduction ASCVD composite	20% reduction MACE composite (CV death, MI, stroke); similar benefits in those with and without DM; greater benefits in those with MIs <2 years, multiple MIs, high-risk features, and PAD
ODYSSEY	(14)	ACS <1 year	Alirocumab	Placebo	3.1 years	15% reduction ASCVD composite	12% reduction CHD events; 15% reduction total mortality
SPIRE 1 and 2	(15)	ASCVD and High Risk	Bococizumab	Placebo	28 months	ASCVD composite 1% (ns) reduction in SPIRE 1 and 21% (*p* = 0.02) reduction in SPIRE 2	Composite of MI, stroke, CV death: 3% (ns) increase in SPIRE 1 and 26% (*p* = 0.007) reduction in SPIRE 2

* *On background statin therapy*.

## What is the Role of PCSK9 Monoclonal Antibody Therapy on Reducing or Retarding Atherosclerosis? The GLAGOV Trial

Prior clinical studies evaluating the impact of statin therapy on change in percent atheroma volume (PAV) from serial intravascular ultrasound (IVUS) studies demonstrated a generally linear relation with the mean achieved LDL-C, with a suggestion of halting of progression of atherosclerosis at LDL-C levels below 2.1 mmol/L (80 mg/dl) (16–20). Only the ASTEROID non-randomized trial (18) involving rosuvastatin brought on-treatment LDL-C levels to below 1.8 mmol/L (70 mg/dL) (mean achieved LDL-C 1.6 mmol/L [60.8 mg/dL]) and demonstrated modest, but significantly significant regression of atherosclerosis (1% reduction in PAV).

In the GLAGOV multicenter, double-blind, placebo-controlled randomized trial (4), 968 patients with angiographic coronary artery disease and an LDL-C of 2.1 mmol/L (80 mg/dL) or higher (or 1.6–2.1 mmol/L [60–80 mg/dL] with 1 major or 3 minor cardiovascular risk factors) on a stable statin dose were enrolled and randomized to evolocumab 420 mg once a month or placebo for 76 weeks. Enrolled subjects underwent IVUS at baseline and follow-up to evaluate the impact of PCSK9 inhibition plaque progression/regression. On-treatment LDL-C was 2.4 mmol/L (93 mg/dL) in placebo group compared to 0.95 mmol/L (37 mg/dL) in the intervention arm. There was a significant difference in the primary outcome of PAV which increased by 0.05% in the placebo group and decreased by 0.95% with evolocumab, *p* < 0.001; similarly, significant differences were also seen in normalized total atheroma volume. Overall, plaque regression was seen in 64.3% of evolocumab treated patients compared to 47.3% of subjects in the control group. In an exploratory analysis, there was an observed inverse linear relation of the extent of change in plaque according to the on-treatment LDL-C down to 0.5 mmol/L (20 mg/dL) without any evidence of a threshold effect. This study was important in demonstrating that further reductions in LDL-C to historically low levels provided additive plaque regression. It is important to realize, however, that while the plaque regression associated with PCSK9 inhibition was statistically significant, the absolute decrease in atheroma volume was quite modest. Thus, the important question remained - would the incremental plaque regression seen with a PCSK9 inhibitor on top of statin therapy ultimately translate into improved cardiovascular outcomes?

## Cardiovascular Outcomes Trials with PCSK9 Inhibitors

Beyond the trials testing the LDL-C lowering efficacy, safety, and impact on atherosclerosis, each of the individual PCSK9 inhibitors has been evaluated in the context of large, dedicated cardiovascular outcomes trials. FOURIER was the first randomized controlled cardiovascular outcomes trial to report out in March 2017. In FOURIER, 27,564 subjects with established ASCVD with additional risk factors and LDL-C of at least 1.8 mmol/L (70 mg/dL) on optimized statin therapy were randomized to evolocumab 140 mg every 2 weeks or 420 mg monthly or matching placebo for a median follow-up of 2.2 years (5). The primary endpoint was a composite of cardiovascular death, myocardial infarction, stroke, hospitalization for unstable angina, or coronary revascularization. Subjects randomized to evolocumab achieved a median LDL-C of 0.78 mmol/L (30 mg/dL) at 48 weeks compared to 2.4 mmol/L (92 mg/dL) in those allocated to placebo. At the end of the trial there was a 15% relative risk reduction (hazard ratio [HR] = 0.85, 95% confidence interval [CI] = 0.72–0.92, *p* < 0.001) in the primary composite endpoint in the evolocumab group relative to placebo (9.8% vs. 11.3% after a median of 2.2 years of follow-up randomized to treatment or placebo among all participants) and a 20% relative risk reduction in the secondary composite endpoint of cardiovascular death, myocardial infarction, and stroke. For the 2.2-year follow-up of the trial this translated to a number needed to treat (NNT) of 66. With the exception of a higher incidence of injection-site reactions associated with evolocumab (2.1 vs. 1.6%), other adverse events, including new-onset diabetes and neurocognitive events, were similar. Other secondary outcomes were also significantly lower in the evolocumab compared to placebo group, including incident myocardial infarction (27% relative risk reduction), stroke (21% relative risk reduction), and coronary revascularization (22% relative risk reduction). However, the incidence of cardiovascular or all-cause death was not significantly different between groups. A more recent analysis of total incident events further confirmed the benefit seen with the primary composite endpoint of initial events; while there was a 15% relative risk reduction seen with the primary composite endpoint, additional events have occurred with a greater difference between groups (26% lower in the evolocumab group), for an overall 18% reduction in risk (RR = 0.82, 95% CI = 0.75–0.90, *p* < 0.001) for total events (6). The authors also described these results in terms of number needed to treat (NNT) and noted that for every 1,000 patients treated with evolocumab for 3 years, compared to placebo, while there would be 22 fewer first events in the evolocumab group (NNT = 45), 52 total events would be prevented (NNT = 19). Analyses of total events, such as this one, provide much more insight into the potential benefits of a therapy, in this case, evolocumab, and should be routinely included in assessment of a new therapy.

Among a subgroup of patients from FOURIER, the EBBINGHAUS substudy involved prospective evaluation of cognitive function (7) using the Cambridge Neuropsychological Test Automated Battery. The primary endpoint was the score on the spatial working memory strategy index of executive function and secondary endpoints included scores for working memory, episodic memory, and psychomotor speed. Among 1,204 patients followed for a median of 18 months, there were no significant differences in either the primary or secondary cognitive endpoints, suggesting that dramatic LDL-C lowering with evolocumab did not impact cognitive function, at least over the short term (19-month treatment observation). Of course longer term follow-up of patients taking PCSK9 inhibitors will be required to allay concerns related to cognitive function in the context of chronic, significant reductions in LDL-C.

The ODYSSEY OUTCOMES trial (14) enrolled patients with a recent acute coronary syndrome (ACS) (myocardial infarction or unstable angina within 1–12 months of enrollment) who were on a high intensity statin for at least 2 weeks with an LDL-C of ≥1.8 mmol/L (70 mg/dL), non-HDL-C of ≥2.6 mmol/L (100 mg/dL) or apolipoprotein B of ≥0.0016 mmol/L (80 mg/dL). Subjects were randomized to alirocumab or placebo, administered every 2 weeks, aiming for a target LDL-C range of 0.65–1.3 mmol/L (25–50 mg/dL); if LDL-C dropped below 0.65 mmol/L (25 mg/dL) the dosage was reduced, and if below 0.4 mmol/L (15 mg/dL), therapy was discontinued. While the intervention group reached an LDL-C of 1.0 mmol/L (38 mg/dL) after 4 months from randomization, this titration scheme resulted in an on-treatment LDL-C of 1.4 mmol/L (53 mg/dL) after 48 months, compared to 2.6 mmol/L (101 mg/dL) in the placebo group (for a 54.7% net reduction). At the end of the treatment period (mean follow-up 3.3 years) there was a 15% relative risk reduction (HR = 0.85, 95% CI 0.78–0.93, *p* = 0.0003) in the primary composite endpoint of CHD death, non-fatal MI, ischemic stroke, or unstable angina requiring hospitalization. The individual endpoints of non-fatal MI, ischemic stroke, and unstable angina, but not CHD death were reduced significantly as well (relative risk reductions of 14, 27, 39%, respectively). Hierarchical testing showed secondary endpoints of CHD events (CHD death, non-fatal MI, unstable angina requiring hospitalization, or ischemia driven coronary revascularization), major CHD events (CHD death or non-fatal MI), CV events (CVD death, non-fatal CHD event, non-fatal ischemic stroke), and death, MI, and ischemic stroke to be reduced significantly as well, but not CHD or CV death separately. However, total mortality was significantly reduced by 15% (*p* = 0.026), but this result should be treated with caution due to the failure of CHD and CV death to be significant (for a specific endpoint, hierarchical testing requires all endpoints listed before the one of interest to be significant). Adverse events, with the exception of injection site reactions (3.8% in the alirocumab and 2.1% in the placebo groups) did not differ between alirocumab and placebo groups. The results of ODYSSEY OUTCOMES further emphasize the potential utility of PCSK9 inhibition for reducing ASCVD events in high risk patients with ACS. It is conceivable that, in the future, PCSK9 inhibition may be part of the medical management of patients presenting with ACS. Dedicated studies aimed at addressing the effectiveness of this approach will be required.

**A** third PCSK9 monoclonal antibody, bococizumab, was under development in the SPIRE program. Both the SPIRE 1 and SPIRE 2 outcomes trials (15) demonstrated a continuous attenuation of LDL-C lowering through 28 months of follow-up in those subjects randomized to bocozumab. The development of antidrug antibodies over time was due to the fact that bococizumab is a humanized (~97% human) but not fully human therapeutic monoclonal antibody and was the likely cause of the attenuation of the LDL-C lowering effect. The SPIRE 1 and SPIRE 2 cardiovascular outcomes trials were stopped early and all further clinical development of the product was halted in November 2017. In the SPIRE-1 cardiovascular outcomes trial which enrolled patients with ASCVD with a baseline LDL-C of ≥1.8 mmol/L (70 mg/dL) showed at discontinuation of the trial no significant differences between bococizumab and placebo groups in primary major adverse cardiovascular events (MACE) composite endpoint of non-fatal myocardial infarction, non-fatal stroke, hospitalization for unstable angina requiring urgent revascularization, or cardiovascular death (HR = 0.99, 95% CI = 0.80–1.22, *p* = 0.94) after a median follow-up of 7 months. However, SPIRE 2 which required a baseline LDL-C of 2.6 mmol/L (≥100 mg/dL) actually did show a significant benefit (HR = 0.79, 95% CI = 0.65–0.97, *p* = 0.021) after a median follow-up of 12 months. However, with the continued attenuation of the LDL-C lowering effect, it is likely that this benefit would have diminished over time. Nonetheless, it is remarkable and informative to note, despite the attenuation in LDL-C lowering with bococizumab, that patients with higher baseline LDL-C (≥100 mg/dL) derived significant reductions in major adverse cardiovascular events, even in the short-term (see below). This finding, coupled with the subgroup analysis from ODYSSEY OUTCOMES, suggests that optimal candidates for PCSK9 inhibitor treatment would be those who fail to reduce LDL-C to below 100 mg/dL despite therapeutic lifestyle changes and maximally tolerated lipid lowering therapies (e.g., statins, ezetimibe).

## Implications of the Cardiovascular Outcomes Trials on Patient Care

The primary results of the cardiovascular outcomes trials are largely consistent and suggest that the addition of PCSK9 inhibitors to background statin therapy in high risk patients is advantageous. Nonetheless, questions remain regarding the patient subgroups that are most likely to benefit from this therapy. Fortunately, several additional analyses have been performed, primarily from FOURIER, that help to answer this question. Of interest, an exploratory analysis demonstrated a linear relationship between achieved LDL-C levels and cardiovascular event rates down to an LDL-C of ~0.5 mmol/L (20 mg/dL), without any evidence of a threshold LDL-C for which no further benefit would be seen (5). This extremely low LDL-C level was associated with a cumulative event rate of 7.5% in the composite endpoint. While these results are provocative, they are also in a subgroup so must be treated with caution, and do not prove that event rates would remain as low over the long-term. Furthermore, while a modest 16% relative risk reduction in the secondary endpoint was noted following randomization during the first year of treatment with evolocumab compared to placebo, a landmark analysis demonstrated a 25% relative risk reduction after follow-up beyond the first year of treatment (5). This observation supports the notion that cardiovascular risk reduction is both a function of extent of LDL-C lowering and duration of therapy, consistent with what has previously been observed with the statin trials (21). Extrapolating the FOURIER findings to perhaps 5 years of treatment may yield absolute risk reductions of about 3.3% or a number needed to treat of 30 for evolocumab compared to placebo, as has been previously suggested (22), comparable to what would be estimated based on statin trials with similar follow-up. Nevertheless, such extrapolation of presumed benefit beyond the actual duration of the trial is speculative and any interpretation must be treated with caution.

In a subgroup analysis comparing subjects with diabetes at baseline vs. those without diabetes, (8) both groups exhibited similar reductions in LDL-C in the evolocumab treated group (57 and 60%, respectively), and there was a similar impact of the treatment on the primary endpoint of cardiovascular death, myocardial infarction, stroke hospitalization for unstable angina, or coronary revascularization (HRs of 0.82, *p* = 0.002 and 0.78, *p* = 0.0002, in those with and without diabetes, respectively). Moreover, both groups demonstrated an identical 2% absolute risk reduction in events translating to an NNT of 50. Importantly, in those without diabetes or with prediabetes at baseline, there were no significant differences in incident diabetes (HRs of 1.05 and 1.00, respectively, n.s.). The more favorable NNT in those who additionally have diabetes is consistent with this being an especially high risk group. Important for the future would be to establish the efficacy of PCSK9 mAb therapy in those with diabetes (especially in those with significant risk factor burden where risk may be similar to many with established ASCVD) for the primary prevention of ASCVD events; however, it is not clear whether such trials will be done in this population.

When stratifying according to baseline LDL-C (<1.8 mmol/L (70 mg/dL) vs. ≥1.8 mmol/L (70 mg/dL), there was no difference in the effect of evolocumab (HRs of 0.80 and 0.86 for the primary endpoint, and 0.70 and 0.81, respectively, for the secondary endpoint, interactions terms not significant between LDL-C groups) (9). There were also no differences in the primary or secondary composite outcomes when comparing those on high-intensity vs. non-high-intensity statin therapy (HRs of 0.85 and 0.85 for the primary endpoint and 0.78 and 0.81, respectively, for the secondary endpoint, interaction terms not significant). The FOURIER investigators also demonstrated a continuous trend for increasing benefit according to achieved LDL-C, with HRs (compared to the reference group of 100 mg/dl or greater) of 0.90, 0.87, 0.75, and 0.69 for those who achieved LDL-C's of 1.8–2.6 mmol/L (70–100 mg/dL), 1.3- <1.8 mmol/L (50–69 mg/dL, 0.5–1.3 mmol/L (20–49 mg/dL), and <0.5 mmol/L (20 mg/dL) (10). Additionally, there were no significant differences in side effects according to achieved LDL-C. Of interest, among the patients who achieved LDL-C <0.26 mmol/L (10 mg/dL) at 4 weeks, there was a 31% relative risk reduction (HR = 0.69, *p* = 0.03) compared to those whose LDL-C was ≥2.6 mmol/L (100 mg/dL), again without any differences in overall adverse events or adverse events requiring discontinuation of therapy. These data appear to suggest that relative benefit from PCSK9 mAb therapy may be both a function of the starting LDL-C level, as well as the achieved LDL-C, with the most benefit seen in those with higher starting LDL-C levels, but also who achieve the lowest on-treatment LDL-C levels.

In Odyssey Outcomes (14) pre-specified subgroups according to baseline LDL-C (<2.1 mmol/L [80 mg/dL], 2.1- <2.6 mmol/L [80- <100 mg/dL], and ≥2.6 mmol/L [100 mg/dL]), those randomized to alirocumab vs. placebo showed a significant reduction in the primary endpoint of 24% (HR = 0.76, 95% CI = 0.65–0.87) only in those with LDL-C ≥2.6 mmol/L (100 mg/dL), although the test for heterogeneity showed the efficacy did not differ significantly across LDL-C groups (*p* = 0.09). Similarly, all-cause mortality also benefitted significantly only for this group (HR = 0.71, 95% CI = 0.56–0.90), but also with no significant differences between LDL-C groups (p-interaction = 0.12). This is also consistent with the SPIRE 2 trial involving bococizumab, which despite its early termination, showed a benefit among those with baseline LDL-C ≥2.6 mmol/L (100 mg/dl) that was not seen in SPIRE 1 whose baseline LDL-C ≥1.8 mmol/L (70 mg/dL) (15).

A numerically greater reduction in the secondary endpoint of cardiovascular death, MI, or stroke was observed in those randomized to evolocumab treatment if the qualifying MI was <2 years prior to enrollment in the trial (24% relative risk reduction) compared to ≥2 years ago, although the interaction term was not significant (*p* = 0.18). Similarly, there tended to be a greater reduction in risk in those who had a history of more than 2 prior MIs compared to a single prior MI, although these differences were also not significant (21vs. 16% relative risk reduction, *p*-interaction = 0.57). Finally, those with multivessel disease demonstrated a greater reduction in risk compared to those without multivessel disease (30 vs. 11% relative risk reductions, p-interaction = 0.03), and a trend toward greater relative risk reduction was seen in those with at least 1 high risk cardiac feature (defined as either being within 2 years from their qualifying MI, having at least two prior MIs, or residual multivessel coronary disease) compared to no high risk features (22 vs. 6% relative risk reduction, *p*-interaction = 0.11) (11). Risk reductions were similar regardless of whether the MI was an ST segment elevation or non-ST segment elevation MI (HR 0.64 and 0.77, both *p* < 0.001) (12).

Perhaps most striking are the outcomes in patients with peripheral arterial disease Subjects with PAD (*n* = 3642) exhibited a 27% relative risk reduction in the composite endpoint of CV death, MI, or stroke, compared to a more modest 19% relative risk reduction in those without PAD, although the interaction for this difference was not significant (*p* = 0.41) (13). Among all patients in FOURIER, there was a substantial 42% relative risk reduction (*p* < 0.01) in major adverse limb event (acute limb ischemia, major amputation, or urgent revascularization).

Consistent with these greater benefits in such high risk patient subgroups, of interest, the recently released 2018 AHA/ACC/AACVPR/AAPA/ABC/ACPM/ADA/AGS/APhA/ASPC/NLA/PCNA Guideline for the Management of Blood Cholesterol has recommended the use of PCSK9 inhibitors for the subgroup of ASCVD patients defined to be at very high risk based on the presence of multiple major ASCVD events (such as both a recent ACS and symptomatic peripheral arterial disease) or a single major event and multiple other high risk conditions (such as diabetes and prior revascularization) or primary severe hypercholesterolemia once maximal other LDL-C lowering therapy with a statin and ezetimibe has been provided (or after statin alone although with less cost-effectiveness noted, at least based on mid-2018 pricing) (23).

Overall, the 3 randomized cardiovascular outcomes trials with PCSK9 inhibitors are instructive and several major lessons can be learned from them (Table 2). First, the type of antibody used to inhibit PCSK9 matters greatly. Humanized antibodies against PCSK9 are immunogenic and are associated with injection site reactions, the development of neutralizing antibodies, and an attenuated LDL-C lowering effect. Second, consistent with the ”lower is better for longer” hypothesis, clinical benefits with alirocumab and bococizumab were greater in those patients who had higher baseline LDL-C. Third, although these trials support the LDL hypothesis of “lowest is best,” the overall cardiovascular risk reduction afforded by dramatic (~60%) LDL-C lowering was modest, possibly because the full benefit of these therapies were not realized over the limited duration of the trials. Fourth, and not surprisingly, the magnitude of absolute benefit is the greatest among the highest risk patients and maximized with longer exposure to drug.

**Table 2:** Lessons learned from the PCSK9 monoclonal antibody outcomes trials.

• Humanized antibodies (bococizumab) against PCSK9 are immunogenic and are associated with injection site reactions, the development of neutralizing antibodies, and an attenuated LDL-C lowering effect.
• Fully human monoclonal antibodies (alirocumab, evolocumab) tend to have a more consistent LDL-C lowering effect.
• Clinical benefits with alirocumab and bococizumab were greater in those patients who had higher baseline LDL-C.
• The PCSK9 monoclonal antibody trials provide evidence supporting the LDL hypothesis that the lowest LDL-C achieved is best.
• The overall cardiovascular risk reduction afforded by dramatic (~60%) LDL-C lowering with PCSK9 inhibition was modest, possibly because the full benefit of these therapies were not realized over the limited duration of the trials.
• The magnitude of absolute benefit is the greatest among the highest risk patients and maximized with longer exposure to drug, suggesting these are important considerations for the use of such agents.

## Conclusions and Interpretation

The results of the recent PCSK9 inhibitor cardiovascular outcomes trials have dramatically changed the landscape regarding the clinical importance of LDL-C reduction and residual ASCVD risk management. The GLAGOV trial leveraged IVUS to demonstrate that the extreme LDL-C lowering with evolocumab on top of statin therapy was associated with regression of atherosclerosis in two-thirds of patients and forecasted the results of the event driven studies. The FOURIER trial was the first of the large outcomes trials to be published and demonstrated a 15% reduction in MACE with a suggestion of greater benefit with lower achieved LDL-C achieved, down to below 0.26 mmol/L (10 mg/dL) without evidence of increased serious adverse events or other safety concerns, at least from the limited 2.2-year median follow-up in FOURIER. The EBBINGHAUS substudy of FOURIER used robust objective measures of cognitive function and demonstrated no differences in outcomes in subjects allocated to evolocumab or placebo, at least over the limited 2-year treatment period. Clearly longer-term follow-up is needed to establish the continued efficacy and safety (including that for cognitive function) of PCSK9 inhibition and the very low LDL-C levels achieved among many subjects. Subsequent analyses of FOURIER have been helpful in understanding which patient subgroups may benefit the most from this therapy. Findings suggests that those with recent or multiple previous MIs, multivessel disease, multiple high-risk features, and peripheral arterial disease may be optimal candidates for PCSK9 inhibitors. The ODYSSEY Outcomes trial extended these findings and demonstrated that those with a recent ACS (within 1 year) garnered similar benefits in MACE reduction with even a possible benefit in total mortality. Moreover, this trial suggested that individuals with a baseline LDL-C ≥2.6 mmol/L (100 mg/dL) enjoy the greatest risk reduction, and perhaps even a reduction in total mortality. Finally, SPIRE demonstrated the importance of utilizing fully human monoclonal antibodies to antagonize PCSK9 so as to avoid the development of anti-drug antibodies, specifically neutralizing antibodies, and attendant attenuation of the LDL-C lowering effect.

While the development of evolocumab and alirocumab provide important advances in the management of dyslipidemia and ASCVD risk, important issues remain. Firstly, significant residual risk still remains despite the functional elimination of LDL-C from the risk equation. Some of this residual risk may be due to other atherogenic lipid fractions that are not modified with PCSK9 inhibition, high baseline risk of those studied, other risk factors that remain inadequately controlled, and non-modifiable risk due to complex atherosclerotic plaques has been previously suggested (22, 24). Moreover, the potential added benefit of the reduction of lipoprotein(a) levels (reduced by about 25% from evolocumab and alirocumab) on reducing ASCVD risk beyond effect of LDL-C reduction remains unknown. Additionally, it remains to be seen whether the ASCVD benefit noted in these short trials will continue and even increase with longer term use of these drugs, and whether the substantial LDL-C reductions observed in these trials will continue after 5 and perhaps 10 years of treatment. Finally, the long-term safety of the very low LDL-C levels achieved in patients treated with PCSK9 inhibitors, as well as long-term cognitive safety is unknown. Further follow-up from these major outcomes trials, as well as open label follow-up of patients on these drugs, will be important to help answer these questions. Finally, it is important to realize that other strategies for PCSK9 inhibition are currently being explored, including gene silencing techniques (silencing RNA) and vaccination. While the development of PCSK9 inhibition is undoubtedly a major advance in the prevention and management of ASCVD, it remains to be determined which strategy will ultimately prove to be the most efficacious and cost-effective.

## Author Contributions

All authors listed have made a substantial, direct and intellectual contribution to the work, and approved it for publication.

### Conflict of Interest Statement

NW reports research support through institution for other research projects (not preparation of this manuscript) from Amgen, Amarin, Novo Nordisk, and Boehringer-Ingelheim, advisory board participation from Novartis and Amarin, consulting from Astra-Zeneca, and speakers bureau participation from Amarin and Sanofi. MS reports advisory board participation from Esperion and Novartis and is a consultant for Amarin.
